# Shifting Pathways of Stimulant Use Among Individuals With Opioid Use Disorder: A Retrospective Analysis of the Last Thirty Years

**DOI:** 10.3389/fpsyt.2021.786056

**Published:** 2021-12-20

**Authors:** Matthew S. Ellis, Zachary A. Kasper, Stephen Scroggins

**Affiliations:** ^1^Department of Psychiatry, Washington University in St. Louis School of Medicine, St. Louis, MO, United States; ^2^College for Public Health and Social Justice, Saint Louis University, St. Louis, MO, United States

**Keywords:** opioid use disorder, opioids, stimulants, addiction, polysubstance use

## Abstract

**Background:** Stimulant use among individuals with opioid use disorder has recently increased, driven by changes in drug distribution channels. However, our understanding of polysubstance use is often limited by a need to provide targeted treatment to a primary drug of addiction. Yet there is a crucial need to better understand pathways to addiction, and how the use of multiple substances may differ between populations, as well as time periods.

**Methods:** Using a national opioid surveillance system, we analyzed survey data from new entrants to 124 opioid use disorder treatment centers from 2017 to 2020. Age of first use was collected for prescription opioids, illicit opioids, prescription stimulants, crack/cocaine, and methamphetamines. Year of initial use of an opioid or stimulant was calculated and grouped by 5 year blocs, inclusive of initial use starting from 1991 and ending in 2020 (*n* = 6,048).

**Results:** Lifetime exposure to stimulants was 82.5% among individuals with opioid use disorder. Mean age of initiation increased for all drugs in 2016–2020, in particular prescription opioids (22.3 to 31.8). Stimulants were initiating drugs for a substantial proportion of individuals with opioid use throughout the analyzed time period. Those initiating opioid/stimulant use from 1991 to 1995 had a mean average of 6.8 years between first and second drug exposure, which steadily decreased to 1.5 years between exposures in 2016–2020. Sankey plots depict significantly more drug transitions in those initiating use from 1991 to 2000 (65.1% had at least two drug transitions) compared to 2010–2020 (16.0%). Opioid-stimulant use increased over time among racial/ethnic minorities, sexual minorities, and those with an educational attainment of high school or less.

**Conclusion:** These data highlight not only the substantial prevalence of stimulant use among individuals who develop opioid use disorder, but also the variability through which pathways of use occur. Prevention and intervention efforts need to take into account increasing ages of initial drug exposures, demographic shifts in stimulant-using populations, and more rapid drug transitions between opioid and stimulants. But at a broader level, prevention, harm reduction ideology, and addiction medicine needs to take into account the ubiquity of polysubstance use among individuals with substance use disorders.

## Introduction

In 2020, the United States reported the greatest number of overdose fatalities on record, over 93,000 (1). Primarily driven by overdoses involving opioids, this surge occurred amidst a pandemic that resulted in interruptions to addiction medicine and social support (2–5). Over the past two decades, the opioid crisis has led to renewed understandings of addiction prevention and treatment, with a number of federal, state and local policies implemented focusing on mitigating supply-side forces such as guidelines and legislation targeting prescription practices (6, 7), prescription drug monitoring programs (8, 9), and abuse-deterrent formulations (10, 11). As the crisis has persisted, recent efforts have been made to better understand the demand-side of addiction (12–15); not only by understanding motivations tied to co-morbid conditions such as mental health and chronic pain (16–18), but also the unique role that polysubstance use (i.e., use of multiple classes of substances) plays in addiction pathways. Evidence suggests that polysubstance use is widely prevalent among individuals with addiction, particularly those with opioid use disorder (19–21).

In recent years, the use of illicit psychostimulants such as methamphetamine and cocaine have increased, particularly among those using opioids (22–27). Much of this shift is due to changes in market supply forces such as production and distribution. In the early 2000s, efforts by law enforcement agencies focused heavily on halting domestic methamphetamine production, so much so that drug seizures from domestic methamphetamine laboratories reached its lowest point in 2019 (28). However, as localized methamphetamine production decreased, there was a proliferation of synthetically produced substances such as fentanyl from foreign countries. Methamphetamine supply in the United States is now primarily driven by an influx of manufacturers from Latin and South America (28).

As a result of these new and prolific distribution channels, reports of psychostimulant use and overdose have increased markedly in recent years (22–27). These increases are partially attributable to an increased and cost-efficient supply, and identifiable or unidentifiable lacing of one drug with another (e.g., fentanyl laced cocaine, methamphetamine laced fentanyl). However, other motivations for the use of psychostimulants among individuals using opioids have been reported, including: self-management of withdrawal symptoms, particularly if opioids are not available; attaining a synergistic high; and to balance one's self out throughout the day with cyclical use of opioids and stimulants (29).

Although opioids were the largest contributor to overdose fatalities, the use of methamphetamine and cocaine has not been absent throughout the opioid crisis. However, our understanding of polysubstance use has been limited, often the result of a need to provide targeted treatment to a primary drug involved in the biological underpinnings of addiction. Yet there is a crucial need to better understand pathways to addiction, and how the use of multiple substances may differ between populations, as well as time periods.

Drug markets are no less susceptible to secular changes than other institutions; indeed, significant disruptions have occurred in recent years as a result of stricter opioid policies, reductions in domestic methamphetamine laboratories, changes in drug market supplies, and the COVID-19 pandemic (28, 30). However, the extent to which these interdictions have influenced polysubstance use is still largely unknown. Retrospective analyses have been used to demonstrate shifts in opioid pathways, primarily national trends suggesting shifts from prescription opioids to heroin. The purpose of the present study was to conduct a retrospective analysis of stimulant use over the past 30 years to investigate the prevalence of stimulant use, better understand pathways that link opioid and stimulant use, and ascertain potential shifts in opioid and stimulant use over time.

## Materials and Methods

### Sample Development

All participants in this study were obtained through the Survey of Key Informants' Patients (SKIP) Program. Briefly, the SKIP Program is an opioid surveillance program that utilizes a serial cross-sectional survey, and is nested within the broader Researched Abuse, Diversion and Addiction-Related Surveillance (RADARS^®^) System. Treatment centers from across the country are selected based on their ability to treat opioid use disorder, and their willingness to participate in an ongoing study regarding opioid use disorder and its correlates. Following verbal consent to participate, each of these treatment centers (i.e., “key informants”) is supplied with, anonymous paper surveys, each ascribed a unique identifier, and directed to provide one survey to persons (i.e., “patients”) 18 years or older who are newly entering the facility with a primary diagnosis of an opioid use disorder, as defined by DSM-IV or V criteria (depending on the time of survey completion). Patients (hereafter, *respondents*) who agree to participate are given a $20 Wal-Mart gift card for completion of the survey, along with a self-addressed stamped envelope to mail the survey directly to Washington University in St. Louis (WUSTL). All protocols were approved by the WUSTL Institutional Review Board.

The present analysis was developed using data from 7,019 respondents who had entered any one of the 124 regionally distributed treatment centers between 2Q2017 and 4Q2020.

### Opioid and Stimulant Use Over Time

Given the overlap of some of these drugs, both chemically and in illicit drug use, drug strata were delineated by having ever used opioids—consisting of two groups, namely prescription opioids and illicit opioids (i.e., heroin or illicit fentanyl)—and stimulants—consisting of prescription stimulants, crack/cocaine, or methamphetamine. Age of first use was collected for each drug of interest, which acted as a proxy for lifetime use. Prevalence estimates were subsequently calculated. However, with a respondent age range of 65 years, we sought to account for the effect that one's length of lifetime drug use (that is, the difference between age at treatment entry and age at first drug exposure) may have on these estimates, as well as the age of first drug exposure. Utilizing Random Iterative Method (RIM) weighting in IBM SPSS Statistics v28, the weighted adjusted prevalence estimates were equivalent to unadjusted rates.

In connection with the sample-wide variance in length of lifetime drug use, and to attend more closely to drug use patterns that have occurred in recent years, our sample was subsequently restricted to individuals whose first use of any drug began within the last 30 years (i.e., no earlier than 1991), thereby removing 14.4% of the original sample for an analytic sample size (N) of 6,048. Age of first use was used to calculate year of initial drug exposure, which was defined as the earliest year for which one of the five drugs of interest was used by a respondent. Additionally, to illustrate changes in drug use over time, quinquennial groupings were established and defined as the year wherein respondents first used their first drug, whatever the drug may be. In accord with this definition, prevalence estimates, mean ages, and number/types of drugs are all reported as a function of the 5 year bloc within which drug use was initiated.

Following this sketch of initial drug exposure (again, irrespective of drug), respondents were categorized into non-exclusive, drug-specific groupings based on which drug(s) they had used first. Temporal comparisons of the mean age of exposure for each are reported.

### Opioid and Stimulant Pathways and Demographics Over Time

To examine polysubstance use pathways and to evaluate the general influence time has had on drug transitions, we further restricted our sample to be constituted solely of individuals who had ever used opioids and stimulants (*n* = 4,935, 81.6% of N). Among these, years to a drug/drug class transition were calculated based on the respondent's age upon initiating any of the respective drugs, whichever came first, and the age at which a change in drug/drug class was made. Drug-drug transitions included the difference among first using any of the five drug groups; class-class transitions included the difference among first using either of the two drug classes. Protracted comparison of these individuals' demographic characteristics and univariate statistics were developed as well.

### Opioid and Stimulant Drug Transitions

In order to observe temporal differences in ordered pathways of substance use, those who initiated more than one drug in a single year were excluded from the baseline analysis sample as the order of use of more than one drug in a single year could not be discerned (*n* = 883). A comparison of those included vs. excluded for analyses are included in Supplementary Table 1. There were no significant differences between those who initiated use with a single vs. multiple substances in the year of initial drug exposure, with the exception of mean age, which differed by less than a year (multiple = 31.6 vs. single = 32.4), and mean age of initiation, which was slightly, but significantly higher for those excluded form analyses (multiple = 18.4 vs. single = 16.6). For those that remained, drugs were ordered by their age of first use and then the differences between these ages were averaged to determine number of years between drug transitions (i.e., first drug to second drug, second drug to third drug, etc.).

Sankey plots were then created, inclusive of participants who (1) reported initiation and transitions to a single substance and (2) those who initiated substance use between 1991 and 2000 or between 2011 and 2020. Participants were then exclusively stratified into one of two groups based on period of first substance initiation: group 1: 1992–2000 and group 2: 2011–2020. Counts of those transitioning from substance-to-substance were then used to construct a Sankey plot for each period. The years from 2001–2010 were excluded from these analyses in order to provide a more distinct temporal comparison of opioid and stimulant drug transitions.

## Results

### Demographic Characteristics

Table 1 describes the demographic profiles of individuals with opioid use disorder who had lifetime exposure to stimulants by 5 year bloc. The proportion of individuals with opioid-stimulant use significantly decreased among females (65.3 to 45.5%, *p* < 0.001), urban residents (57.7 to 51.5%, *p* < 0.001), and those with an educational attainment of some college (41.2 to 32.4%, *p* < 0.001). Conversely, the proportion of opioid-stimulant users significantly increased among racial/ethnic minorities (20.3 to 36.2%, *p* < 0.001), sexual minorities (11.5 to 27.7%, *p* = 0.001), suburban residents (23.1% to 24.2%, *p* = 0.002), residents of the Western region of the United States (17.2 to 31.9%, *p* < 0.001), and those with an educational attainment of high school or less (54.5 to 58.8%, *p* < 0.001). Lifetime history of prior treatment episodes was endorsed by the majority of respondents in each 5 year bloc, but significantly decreased in the proportion, from 87.6 to 62.3% (*p* < 0.001). Mean age of respondents at the time of survey completion decreased from 40.5 to 32.3 (*p* < 0.001), while mean age of initial drug exposure increased from 15.4 to 25.6 (*p* < 0.001).

**Table 1:** Demographic characteristics of individuals with opioid use disorder with lifetime exposure to stimulant drugs, grouped by year of initial drug exposure.

	**1991–1995 (*****n*** **=** **680)**		**1996–2000 (*****n*** **=** **1,044)**		**2001–2005 (*****n*** **=** **1,399)**		**2006–2010 (*****n*** **=** **1,193)**		**2011–2015 (*****n*** **=** **550)**		**2016–2020 (*****n*** **=** **69)**		**Sig. (X^**2**^)**
**Demographics**
Female	*441*	65.3%	*620*	59.7%	*802*	57.8%	*662*	55.8%	*296*	54.1%	*30*	45.5%	<0.001
Racial/ethnic minority	*138*	20.3%	*205*	19.6%	*227*	16.2%	*236*	19.8%	*160*	29.1%	*25*	36.2%	<0.001
Sexual minority	*38*	11.5%	*53*	11.0%	*78*	11.3%	*77*	13.4%	*56*	18.1%	*13*	27.7%	0.001
Mean age (SD)		40.5 (5.9)		36.2 (5.4)		32.0 (5.2)		28.0 (5.1)		25.7 (6.2)		32.3 (7.2)	<0.001
Mean age of initial exposure (SD)		15.4 (5.6)		15.9 (5.2)		16.6 (4.9)		17.4 (4.9)		19.5 (6.2)		25.6 (10)	<0.001
**Urbanicity**
Urban	*382*	57.7%	*530*	52.0%	*668*	48.8%	*540*	46.6%	*236*	43.7%	*34*	51.5%	<0.001
Suburban	*153*	23.1%	*250*	24.5%	*380*	27.8%	*351*	30.3%	*167*	30.9%	*16*	24.2%	0.002
Rural	*127*	19.2%	*240*	23.5%	*320*	23.4%	*267*	23.1%	*137*	25.4%	*16*	24.2%	0.186
**Regionality**
West	*117*	17.2%	*168*	16.1%	*261*	18.7%	*234*	19.6%	*135*	24.5%	*22*	31.9%	<0.001
Midwest	*187*	27.5%	*270*	25.9%	*369*	26.4%	*325*	27.2%	*131*	23.8%	*17*	24.6%	0.070
Northeast	*76*	11.2%	*139*	13.3%	*214*	15.3%	*172*	14.4%	*66*	12.0%	*4*	5.8%	0.030
South	*300*	44.1%	*467*	44.7%	*555*	39.7%	*462*	38.7%	*218*	39.6%	*26*	37.7%	0.023
**Healthcare coverage**
None	*237*	41.0%	*341*	38.5%	*491*	40.5%	*376*	36.4%	*167*	34.7%	*19*	33.3%	0.104
Covered under another individual	*16*	2.8%	*26*	2.9%	*28*	2.3%	*70*	6.8%	*76*	15.8%	*8*	14.0%	<0.001
Medicare/Medicaid	*282*	48.8%	*440*	49.7%	*604*	49.9%	*512*	49.6%	*206*	42.8%	*24*	42.1%	0.011
Private	*33*	5.7%	*58*	6.6%	*69*	5.7%	*67*	6.5%	*28*	5.8%	*6*	10.5%	0.691
VA/Military healthcare	*10*	1.7%	*20*	2.3%	*19*	1.6%	*8*	0.8%	*4*	0.8%	*0*	0.0%	0.075
Any healthcare coverage	*341*	59.0%	*544*	61.5%	*720*	59.5%	*657*	63.6%	*314*	65.3%	*38*	66.7%	0.104
**Educational attainment**
High school or less	*364*	54.5%	*561*	54.2%	*789*	56.8%	*734*	62.0%	*374*	68.1%	*40*	58.8%	<0.001
Some college	*275*	41.2%	*420*	40.5%	*534*	38.5%	*403*	34.0%	*166*	30.2%	*22*	32.4%	<0.001
Bachelor's or higher	*29*	4.3%	*55*	5.3%	*65*	4.7%	*47*	4.0%	*9*	1.6%	*6*	8.8%	0.006
**Prior OUD treatment episodes**	*595*	87.6%	*888*	85.1%	*1,170*	83.8%	*939*	78.8%	*405*	73.6%	*43*	62.3%	<0.001

### Stimulant Use Among Individuals With Opioid Use Disorder

**Figure 1:**
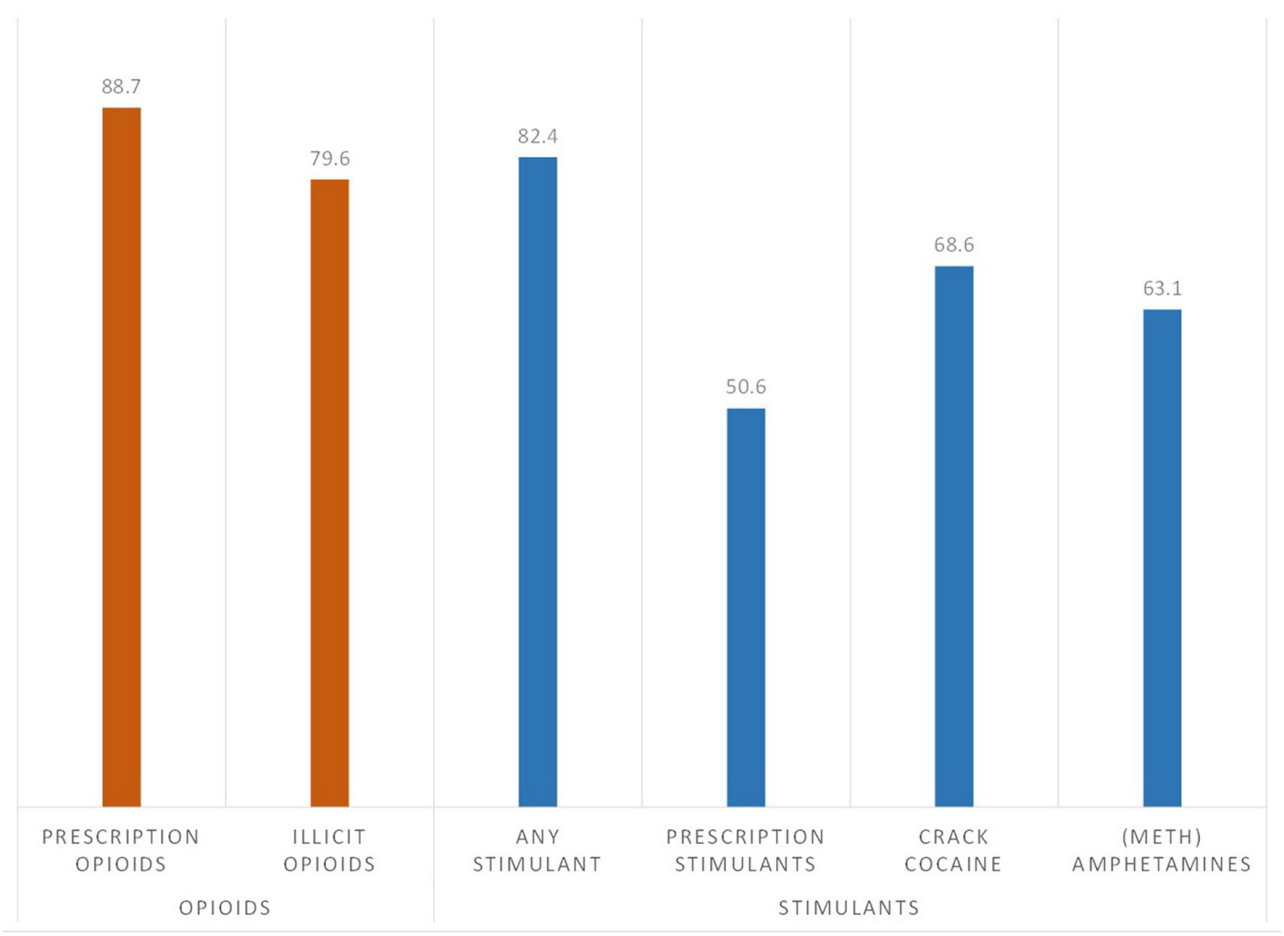
Prevalence of lifetime exposure to opioid and stimulants among individuals with opioid use disorder (*n* = 7,109), adjusted for age of initial drug exposure and time since year of initial drug exposure.

As shown in Figure 1, lifetime exposure to stimulants was very high among this sample of individuals with opioid use disorder, with 82.4% reporting the use of prescription stimulants, crack/cocaine or methamphetamine, after adjusting for time since initial drug exposure to an opioid or stimulant. Crack/cocaine had the highest adjusted rate of lifetime use (68.6%), followed by methamphetamines (63.1%) and prescription stimulants (50.6%).

**Figure 2:**
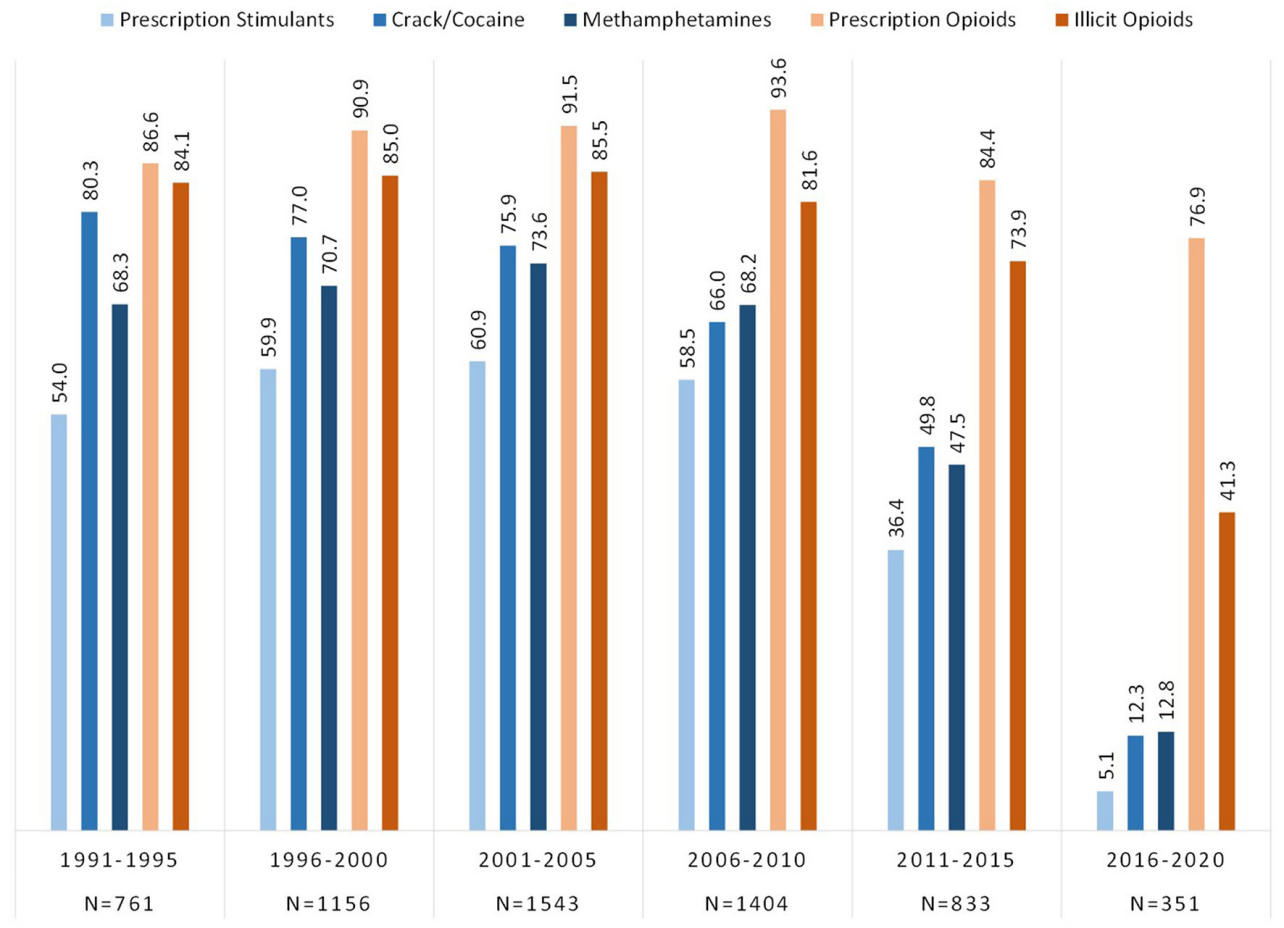
Prevalence of lifetime exposure to opioids and stimulants grouped by year of initial drug exposure among individuals with opioid use disorder.

Respondents were categorized by the year of initial drug exposure to either an opioid or a stimulant and grouped into 5 year blocs across the past 30 years, from 1991 to 2020. Figure 2 depicts lifetime use of opioid and stimulant categories by respondent's year of initial drug exposure. Of those who initiated use from 1991 to 2010, lifetime use of all three stimulant categories were reported by over half the sample; 54.0–58.5% for prescription stimulants, 68.3–68.2% for methamphetamines, and 80.3–66.0% for crack/cocaine. These rates were lower in more recent years with just 5.1% exposed to prescription stimulants, and 12–13% to crack/cocaine and methamphetamines, by 2016–2020.

**Figure 3:**
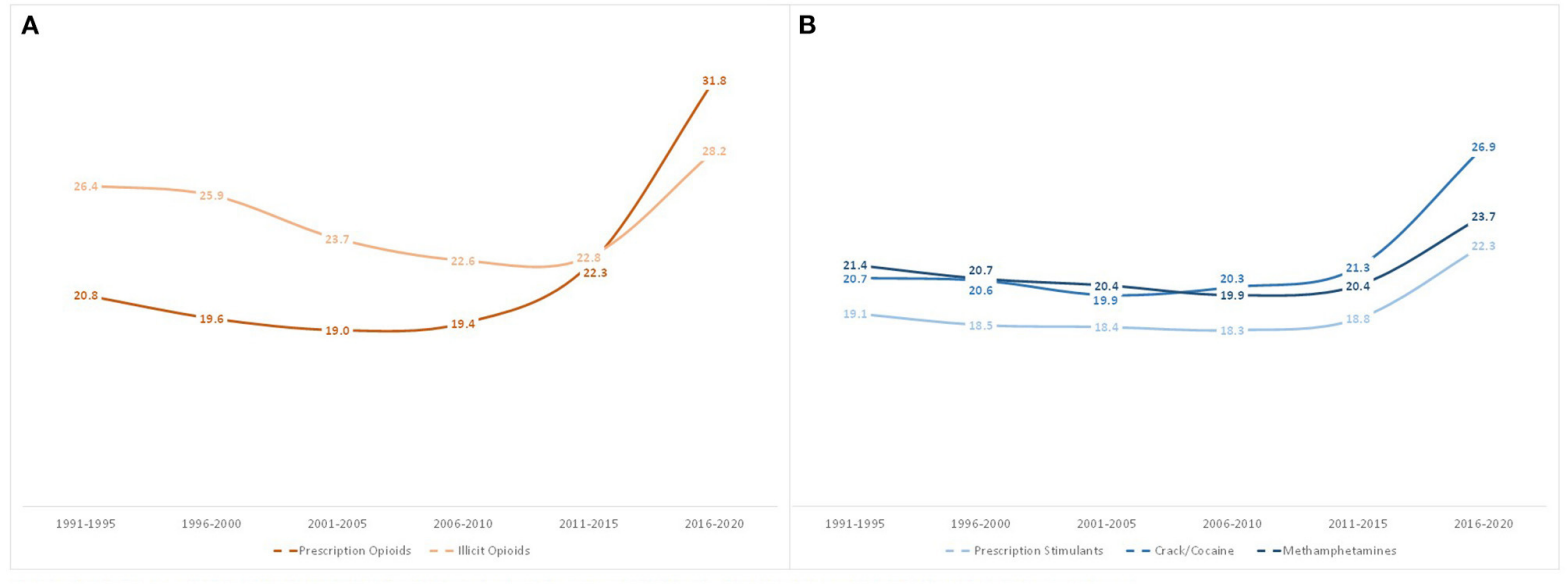
Mean age of first use of opioids **(A)** and stimulants **(B)** among individuals with opioid use disorder by year of drug exposure.

Figure 3 shows that the mean age of initiation for all opioid and stimulant classes ever used stayed relatively stable from 1991 to 2015, but significantly increased in 2016–2020. Age of first exposure to prescription opioids saw the greatest increase, from a mean age of initiation of 22.3 in those first exposed in 2011–2015, to 31.8 for those initiating use in 2016–2020. Similarly, but to a lesser extent, illicit opioid initiation rose from 22.8 to 28.2 years old, crack/cocaine from 21.3 to 26.9 years old, prescription stimulants from 18.8 to 22.3 years old, and methamphetamines from 20.4 to 23.7 years old.

### Initial Drug Exposure

**Figure 4:**
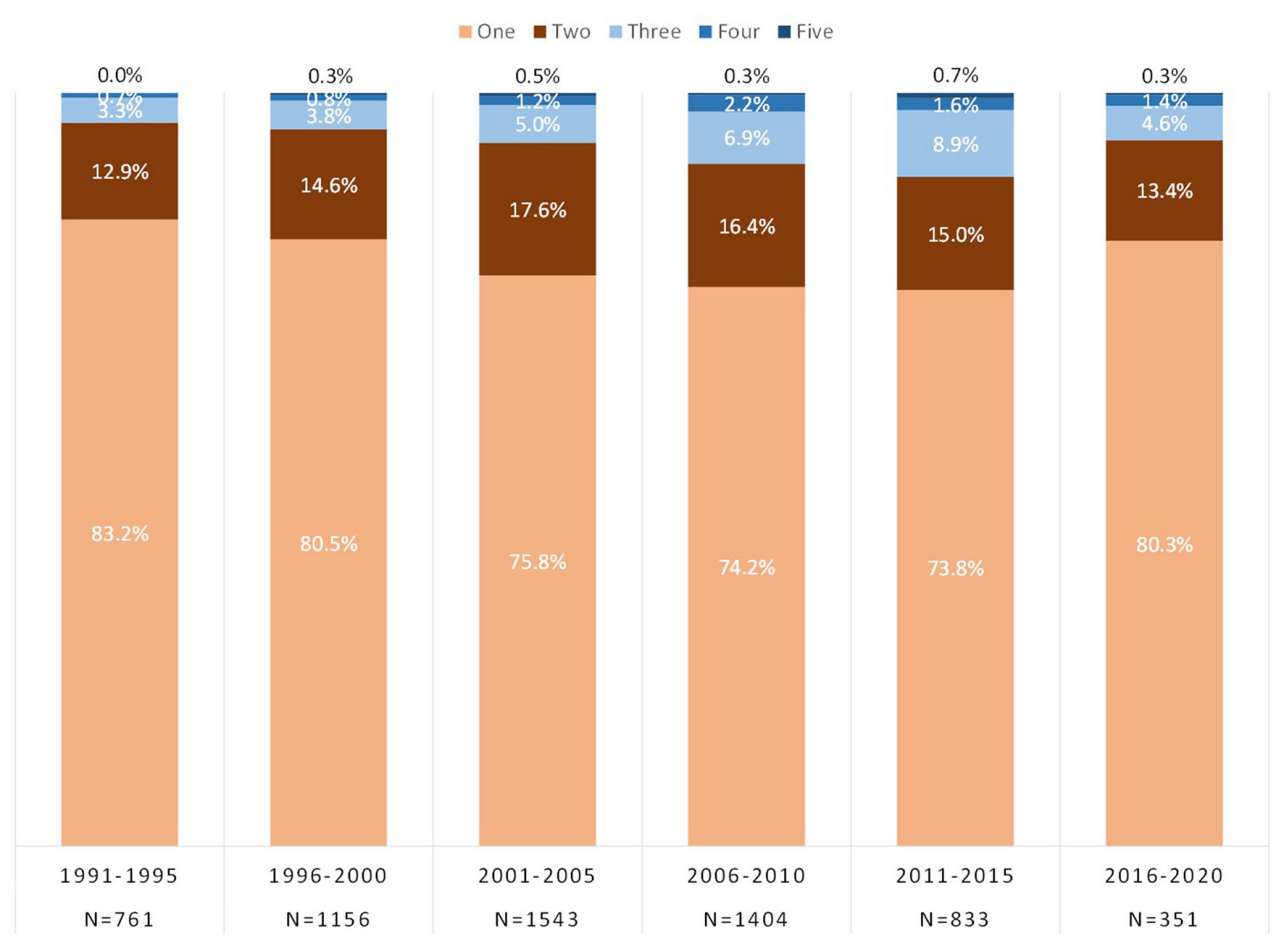
Number of drugs (opioids and/or stimulants) used in the year of initial drug exposure.

**Figure 5:**
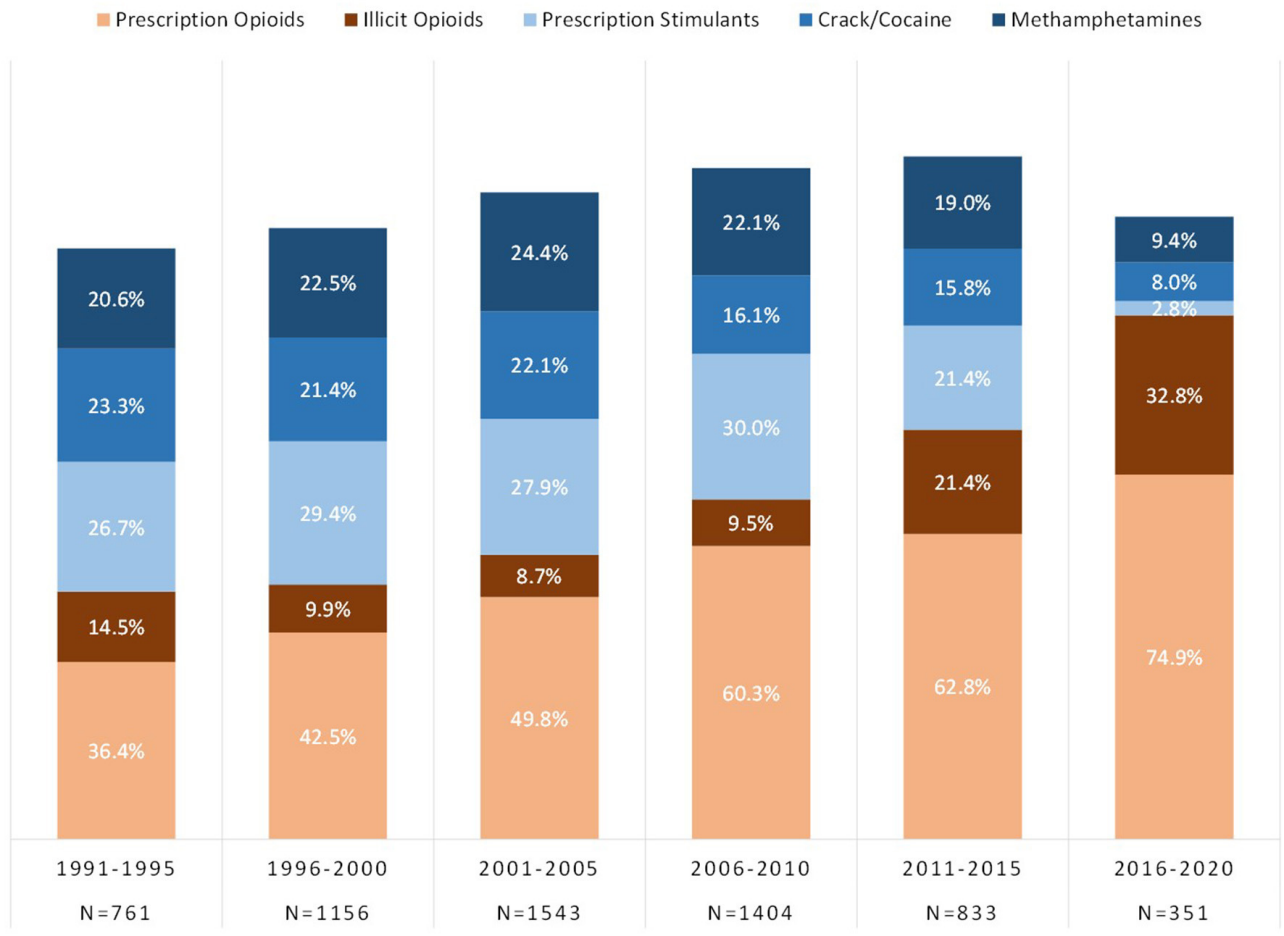
Types of opioids and stimulants used in the year of initial drug exposure. Total percentage may exceed 100% as multiple drugs may have been used in the year of initial drug exposure.

Figure 4 shows the number of drugs respondents were exposed to in the year of initial drug exposure to an opioid (prescription opioids or illicit opioids) and/or a stimulant (prescription stimulants, crack/cocaine, or methamphetamines). Respondents primarily reported the use of a single substance in the year of initial drug exposure, although this decreased from 83.2% in 1991–1995 to 73.8% in 2011–2015, and finally increasing back up to 80.3% in 2016–2020. The specific drugs initiated in the year of initial drug exposure are shown in Figure 5, taking into account the use of multiple drugs in a single year (i.e., totals may equal over 100%). The use of prescription opioids as an initiating drug increased from 36.4% in 1991–1995 to 74.9% in 2016–2020. Illicit opioids as an initiating drug increased from a low of 8.7% in 2001–2005 to 32.8% in 2016–2020. Stimulants were initiating drugs for a substantial proportion of individuals with opioid use disorder in the 1990s and early 2000s, decreasing significantly to the point where <10% reported initiating use with each stimulant class: prescription stimulant as initiating drug decreased from 26.7 to 2.8%, crack/cocaine from 23.3 to 8.0%, and methamphetamine from 20.6 to 9.4%.

**Figure 6:**
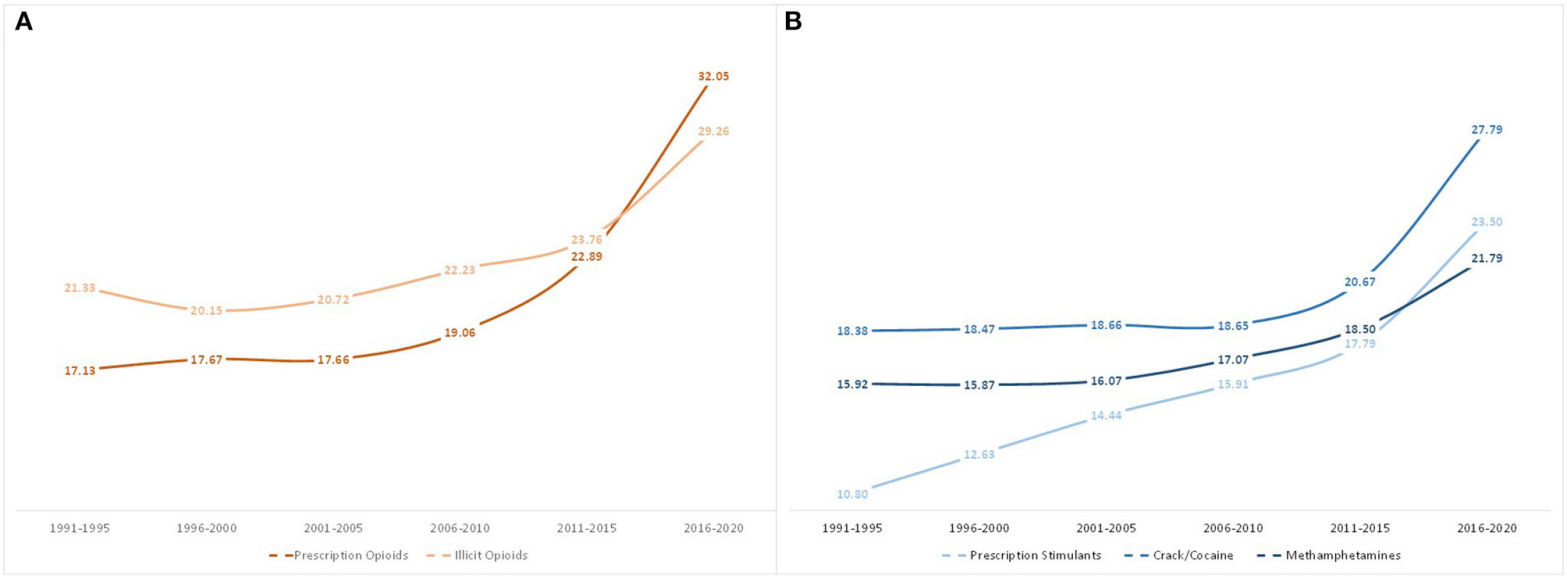
Mean age of first use of opioids or stimulants when used as the initial drug of exposure, grouped by year of initial drug exposure.

Figure 6 outlines the mean age of only one's initial drug of exposure by year of exposure. Similar to Figure 3, the mean age of those initiating their opioid/stimulant use with prescription opioids increased from 17.13 in 1991–1995 to 32.1 in 2016–2020, and illicit opioid initiators had a mean age increase from 21.3 to 29.3. For those who initiated use with stimulants, the largest increase was among prescription stimulant initiators, whose mean age grew from 10.8 in 1991–1995 to 23.5 in 2016–2020. This was followed by crack/cocaine (18.4 to 27.8), and methamphetamines (15.9 to 21.8).

### Drug Transitions and Pathways

**Figure 7:**
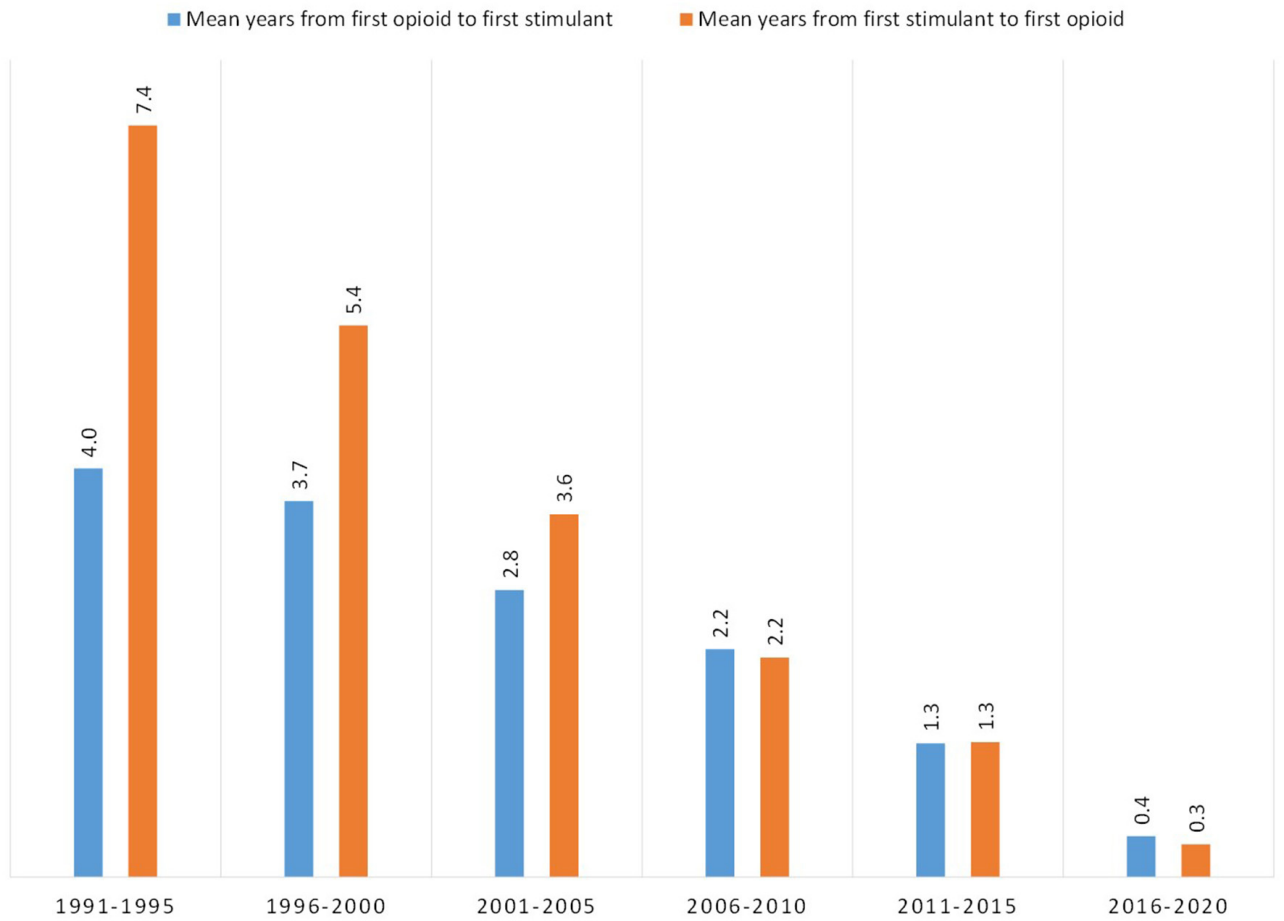
Mean number of years between first use of an opioid and first use of a stimulant, or first use of a stimulant and first use of opioid, grouped by year of initial drug exposure.

Using age of initial exposure, the mean number of years was calculated between a respondent's first use of an opioid and subsequent first use of a stimulant, and vice-versa, depending on the order of use (Figure 7). In 1991–1995, those initiating use of an opioid subsequently initiated use of a stimulant on average, 4 years later, while those initiating use of a stimulant subsequently initiated use of an opioid 7.4 years later. The mean number of years between exposures of opioids and stimulants decreased for both ordered types of respondents over time, although the decrease was more drastic for those transitioning from stimulants to opioids. Those initiating use in 2006–2020 had similar transition times regardless of the pathway, 2.2 years, and these similarly decreased to 1.3 years in 2011–2015 and 0.3–0.4 years in 2016–2020.

**Figure 8:**
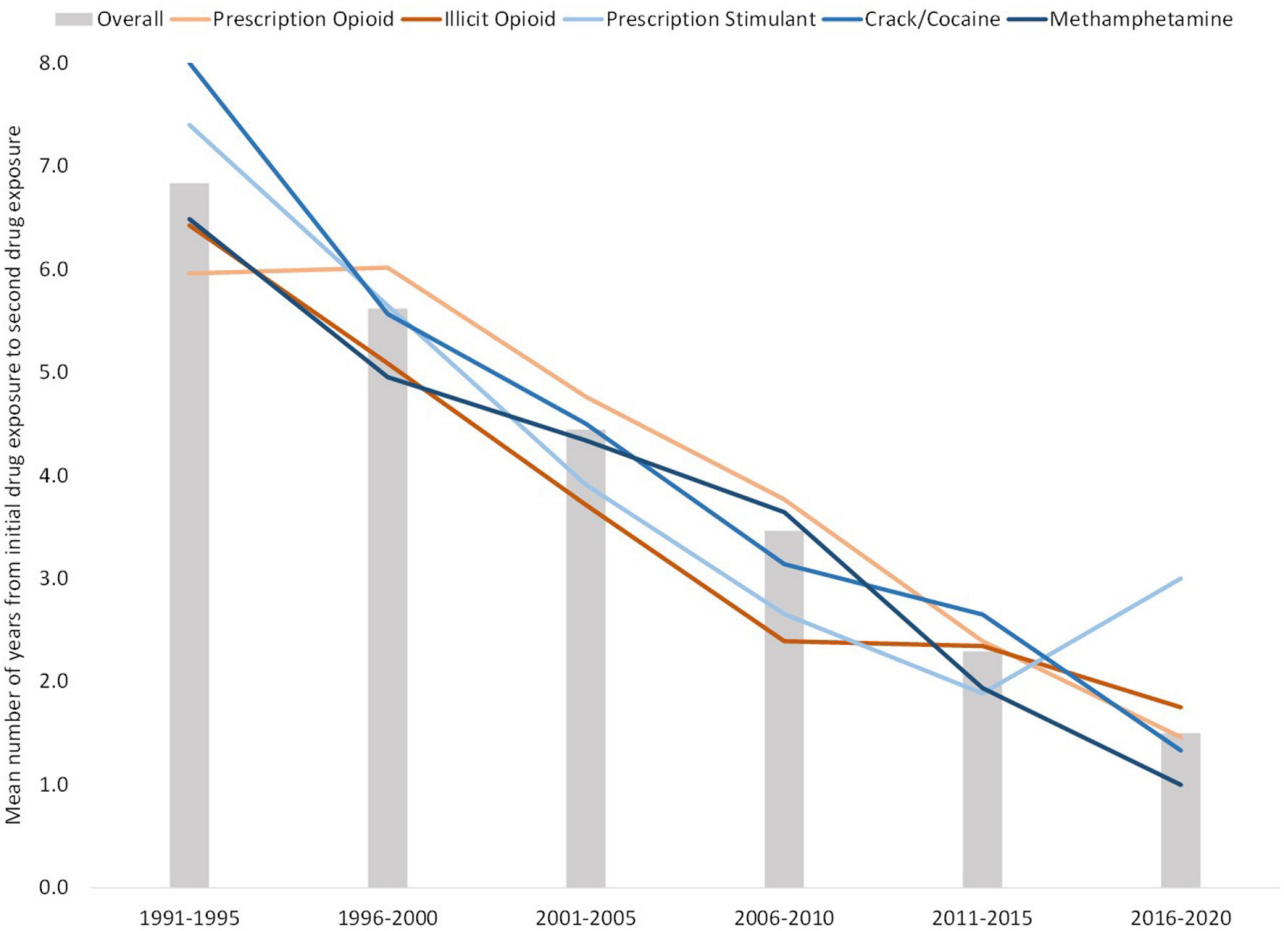
Mean number of years between initial and second drug exposure, grouped by year of initial drug exposure. Bars reflect mean number of years between transitions for all drugs, with lines representing mean number of years between transitions between specific initial drugs and a secondary drug.

**Figure 9:**
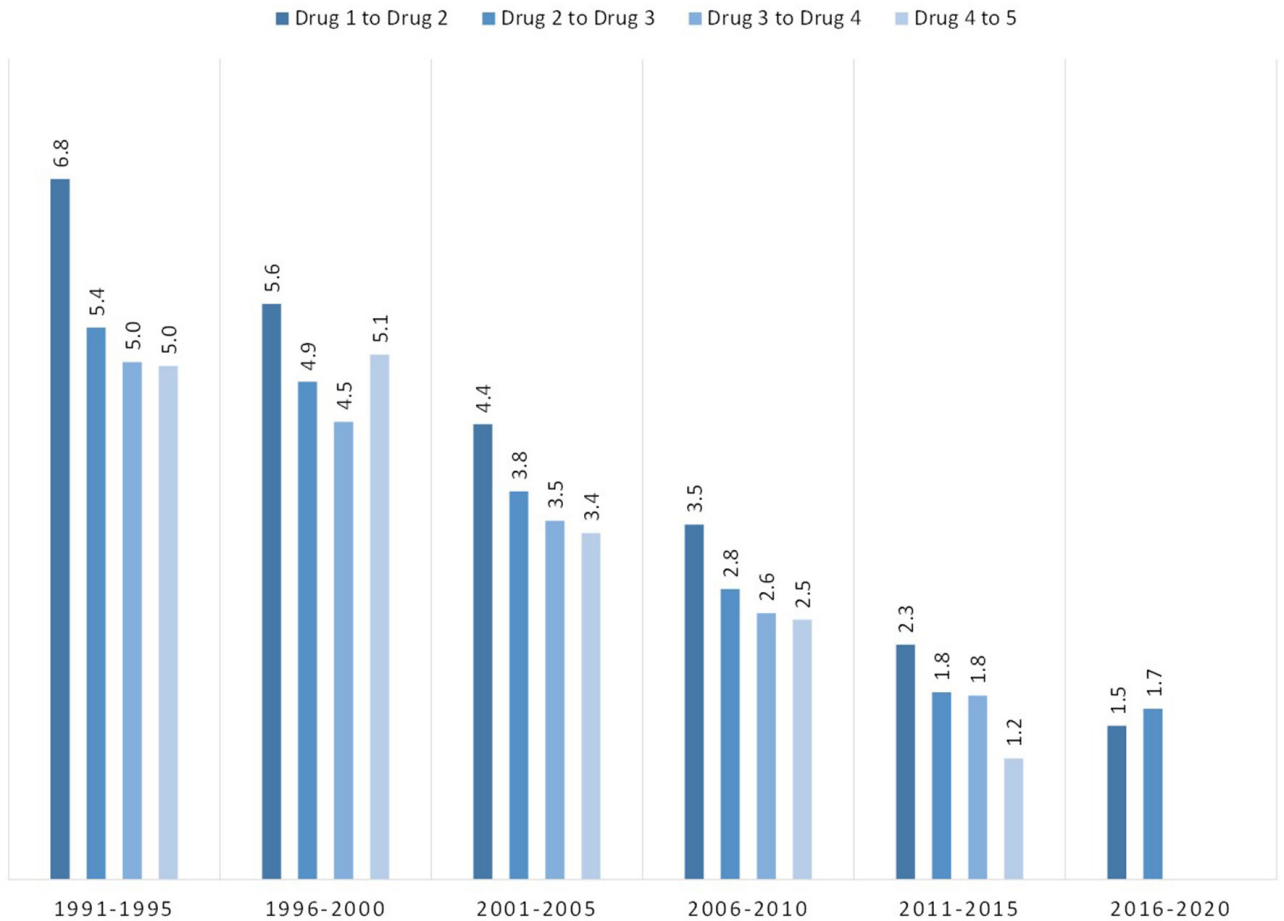
Mean number of years between drug exposures, grouped by year of initial drug exposure.

Excluding individuals who used multiple drugs in a single year to reduce data noise and uncertainty of order of use, 76.7% of respondents had at least one drug transition (i.e., initiated use of another drug in a subsequent year) among the five studied drug categories. Figure 8 shows the mean number of years between initial drug exposure and the first drug transition. Those initiating use from 1991 to 1995 had a mean average of 6.8 years between first and second drug exposure. However, this steadily decreased over time to 1.5 years between exposures among 2016–2020 initiators. As shown in the figure, this trend of decreasing time between drug transitions over each 5 year bloc was consistent regardless of which drug was the initial drug of exposure. This trend also applied to subsequent drug transitions. Of those who had subsequent drug transitions across the five studied drugs, drug transition times decreased slightly after the initial drug transition, but were still relatively similar within their respective 5 year bloc (Figure 9). Of those who initiated use in 1991–1995, mean drug transitions took 5–6 years. This steadily decreased to where, in 2011–2015 transition times were 1–2 years. Initiators in 2016–2020 had only two drug transitions by the time of data analysis, with transition time of 1.5 years.

**Figure 10:**
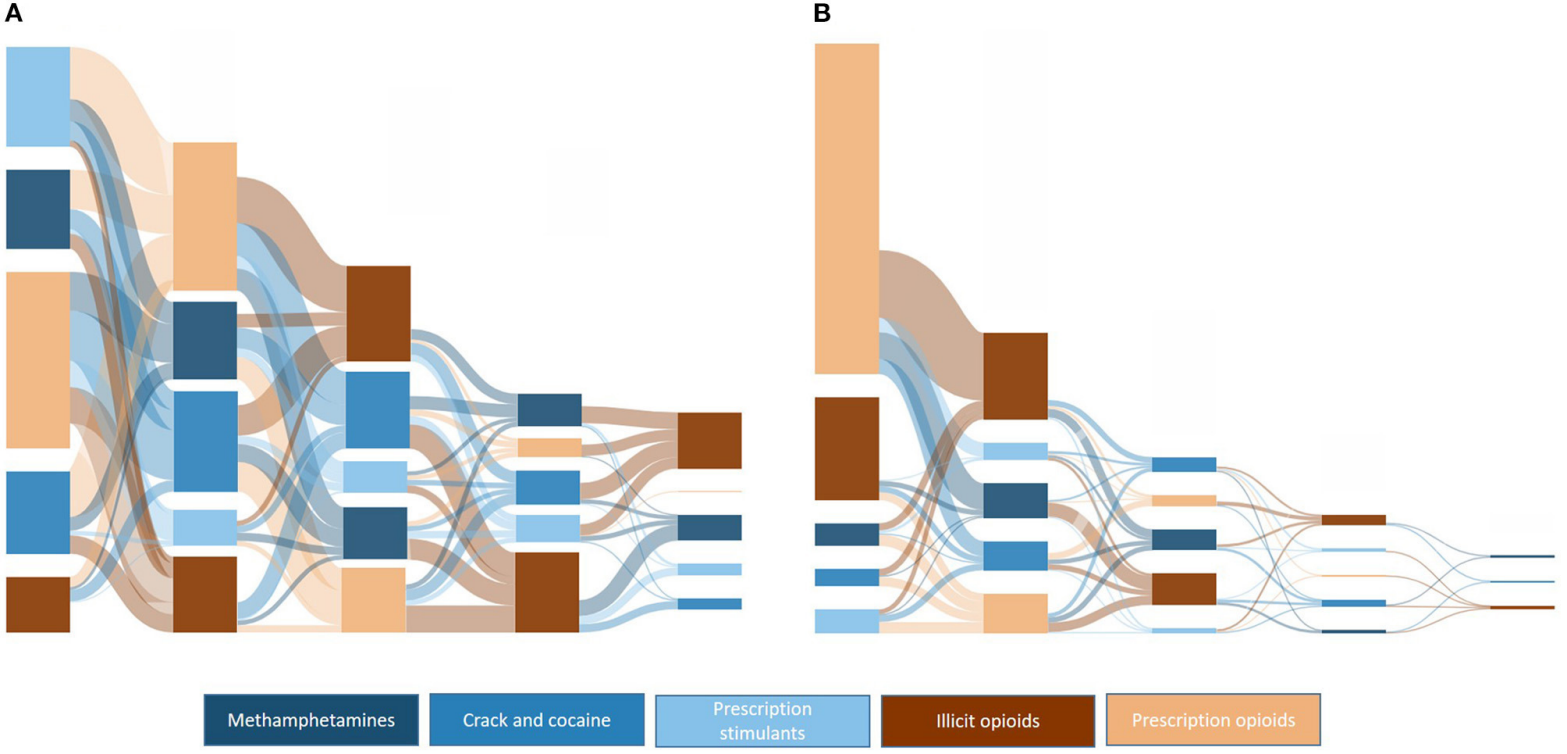
Sankey plots of ordered drug transitions by decade of initial drug exposure: **(A)** 1991–2000, *n* = 1,079; **(B)** 2010–2020, *n* = 749.

Figure 10 provides a comparative overview of drug transitions from 1991 to 2000 and 2010 to 2020 in order to demonstrate temporal shifts in drug transition pathways. As can be seen, there was significant variability in pathways from 1991 to 2000, with 88.8% or respondents having one drug transition, 65.1% with two drug transitions, 39.1% having three drug transitions, and 15.4% having four drug transitions; significantly different from 41.8, 16.0, 5.2, and 1.5%, respectively, in those who initiated use from 2010 to 2020. Notably, those initiating use in the last decade primarily did so through prescription opioids, compared to initiates from 1991 to 2000, who had roughly equal proportions of initiation through stimulants and prescription opioids. Illicit opioids were also more common in later stages of use from 1992 to 2000, compared to earlier stages in 2010–2020.

## Discussion

These data suggest that exposure to stimulant drugs is extremely common among individuals who develop opioid use disorder. Unsurprisingly, this retrospective analysis indicates that stimulant exposure grows in prevalence the longer time has elapsed since initial drug exposure; greater lengths of time likely provide greater opportunities for lifetime exposure. This appears to hold true not just for illicit stimulant drugs, but for prescription stimulants as well. However, there were other notable trends visible over the past 30 years.

Most notably, the time elapsed between exposures of differing drug categories significantly decreased over time. This was visible in both class-class pathways (i.e., opioid-to-stimulant and stimulant-to-opioid) and drug-drug pathways, irrespective of the initiation drug or number of drug transitions. All showed a steady decline, with an average of 5 years between drug exposures in the early 1990s, to a year or less in 2016–2020. The reasons behind this trend likely involves a multitude of factors. First, market forces significantly have changed in the past 30 years. In the early 1990s, the prescription opioid crisis was in its infancy and methamphetamine was often relegated to rural areas through domestic production, likely making access to these drugs scarcer than in ensuing years. As the prescription opioid crisis broadened, subsequent drug production shifted first to cheaper heroin and crack/cocaine, and then to even more cost-efficient synthetic drugs such as fentanyl and methamphetamine, produced through precursors obtained from one foreign country to be produced in another, eventually distributed in the United States (28). As these markets have grown, access and availability have responded accordingly. There is also likely some measure of compensatory use of other drugs when supplies of a preferred drug are limited. Shifts to heroin and methamphetamine have been observed when access to prescription opioids has become more limited as a result of supply-side policies targeting reduction in their distribution (29, 31). It is also possible that exposure has been complicated in recent years by adulterated drugs such as methamphetamine-laced fentanyl or fentanyl-laced cocaine. Current prevention and harm reduction efforts need to take into account evidence that suggests transitions from one drug to another are now occurring at a rapid rate, and will likely increase the rapidity with which oral use may graduate to non-oral use. Indeed, comprehensive care would be amiss if it did not incorporate these factors into the current regimen of naloxone promotion, pre-exposure prophylaxis, needle exchanges and educational efforts.

Prevention and intervention efforts should also take into account the shifting ages at which initiation of use has changed in recent years. Prescription opioid initiation saw the largest increase in age of exposure, increasing from late teens-early twenties to early thirties. For those whose first exposure to an opioid or stimulant was through prescription stimulants, the mean age rose from 10 years old to 23.5 years. These dramatic shifts may be the result of a greater awareness of the potential harms or consequences of these medications among healthcare providers and systems, leading to reductions of prescriptions of opioids and stimulants among younger individuals. This may have resulted in subsequent increases in age of exposure for other drugs that followed initial exposure through a prescription. However, there were significant increases also observed in those whose first opioid or stimulant drug was an illicit one. While further research is needed to understand these shifts, it is possible that prevention and educational efforts targeting young adults have had an effect, and further efforts are needed to target those in their late twenties or early thirties. This is particularly notable in light of the recent pandemic, as well as earlier recessions in the time period of analysis, which caused social and economic upheaval that may disproportionately have impacted individuals in these age groups who are often early in their careers and relationships. In fact, this may help explain other demographic shifts in stimulant use that occurred over time, particularly among those that are often at higher risk of being impacted economically during times of national distress, racial/ethnic minorities, sexual minorities, and those with lower educational attainment.

These data also suggest that opioid and stimulant use occurs across a variety of pathways. However, there do appear to have been shifts in these pathways over time. Interestingly, despite the focus of federal and state policies on mitigating the opioid epidemic in the 2010s, 2016–2020 had the greatest proportion of initiators through opioids, both prescription and illicit. To the latter point, a third of all initiators in 2016–2020 used illicit opioids, further reinforcing the broadening of illicit opioids as one's first experience with opioids, presenting significant dangers to opioid naïve individuals who may be inexperienced in dosing, titration and the presence of admixtures such as fentanyl-laced heroin. In terms of stimulants, since this is a sample of individuals who develop opioid use disorder, it is possible that those who initiated drug use through stimulants have not yet graduated to opioids, and thus have yet to be captured in this sample, leading to lower rates of stimulant initiation than previous years. However, the mean time lapse between transitions would counter this argument as transitions to other drugs are now occurring at a rapid rate. The important point here is that, while not all individuals with opioid use disorder used stimulants, it is notable that throughout the opioid crisis, a substantial number of individuals were exposed to stimulants prior to opioids. This further underscores the complicated nature of polysubstance use. Indeed, the transition plots demonstrate the significant variability of pathways between opioids and stimulants, particularly as time has progressed. Transition plots from 1991 to 2000 showed significant exposure to multiple drugs, but there were few clear-cut pathways that would suggest a commonality of drug pathways or “gateway” drugs, at least within the sphere of stimulants and opioids. Pathways were somewhat easier to discern in more recent years. From 2010 to 2020, the majority of initiators started with prescription opioids and moved to illicit opioids, while those who initiated use with stimulants next went to prescription opioids. This further underscores the need for continued efforts to mitigate diversion of prescription opioids and safe prescribing practices. Despite more rapid transition times in recent years, it is possible, and may be more likely given recent increases in methamphetamine and cocaine use, these individuals will engage in polysubstance use inclusive of a wider variety of drugs as years progress. Treatment for opioid use disorder needs to take into account polysubstance use, viewing addiction as a broader condition that encompasses the use of multiple drugs, rather than a condition isolated to a single, primary drug of use. This includes a deeper understanding of motivations for the use of different classes of substances, particularly the potential for self-management of addiction to one drug with another, and perceptions that the use of other substance outside of one's primary drug of addiction are conceptually different when considering one's addiction.

There are several limitations that are important to note when interpreting these data. First, these data are reflective of individuals entering a treatment program for opioid use disorder within the past 10 years, and thus may reflect a population for which treatment retention and success is lower than average, as well as potentially including a measure of survivor bias, wherein a certain proportion likely succumbed to an overdose or drug-associated fatality. Second, our data are limited in more recent years by a “treatment-gap” bias, wherein there are likely initiators in 2016–2020 whose use has not progressed to the point where treatment is sought, thus potentially reducing generalizability to recreational or non-problematic individuals using opioids. In addition, it is possible that there are significant differences in the time and severity in the escalation of use that drives treatment-seeking behavior, which may limit direct comparisons between 5 year blocs. Third, our data assess lifetime exposure and does not assess duration or severity of use of these drugs, or the use of other substances that may have impacted pathways of stimulant and opioid use such as tobacco, alcohol, marijuana or other substances. Finally, and most notably, these data assess age of exposure, which does not take into account the motivations or reasons behind use. While less applicable to the illicit drugs, it is likely that first exposure to prescription opioids or stimulants were through therapeutic channels, for therapeutic purposes, and therefore, may not be representative of problematic use. However, therapeutic channels as initial exposures to opioids and stimulants have been shown to increase of subsequent problematic use.

These data highlight not only the substantial prevalence of stimulant use among individuals who develop opioid use disorder, but also that opioid and stimulant polysubstance use develops through a number of pathways, often a result of both supply (i.e., accessibility) and demand (i.e., motivations for use) side factors. Importantly, stimulant use played a significant introductory role to substance use prior to opioid initiation, and despite recent increases in national trends of illicit stimulant use, they have played a significant contributing role throughout from the beginning of the opioid crisis. Recent demographic shifts indicate that those initiating use of opioids and stimulants today may be different than those from years past, particularly in young adults of a greater age. Additionally, transitions to other drugs is occurring at a far faster rate than previously seen. Prevention and intervention efforts need to take these shifts into account. But at a broader level, preventative educational and screening efforts, harm reduction ideology, and treatment through addiction medicine needs to take into account the ubiquity of polysubstance use among individuals with substance use disorders.

## Data Availability Statement

The data analyzed in this study is subject to the following licenses/restrictions: Proprietary to the RADARS System. Requests to access these datasets should be directed to ellism@wustl.edu.

## Ethics Statement

The studies involving human participants were reviewed and approved by Washington University in St. Louis Institutional Review Board. Written informed consent for participation was not required for this study in accordance with the national legislation and the institutional requirements.

## Author Contributions

All authors participated in analyzing and interpreting the data and in drafting and reviewing the manuscript.

## Funding

This work was sponsored by the Researched Abuse, Diversion and Addiction-Related Surveillance (RADARS) System, an independent nonprofit post-marketing surveillance system that was supported by subscription fees from pharmaceutical manufacturers who use these data for pharmacovigilance activities and to meet regulatory obligations. Subscribers do not participate in data collection nor do they have access to the raw data. RADARS System is the property of Denver Health and Hospital Authority, a political subdivision of the State of Colorado. ME and ZK are employees of Washington University in St. Louis, which receives research funding from Denver Health and Hospital Authority.

## Conflict of Interest

ME is a member of the Scientific Advisory Group for the National Drug Early Warning System. The remaining authors declare that the research was conducted in the absence of any commercial or financial relationships that could be construed as a potential conflict of interest.

## Publisher's Note

All claims expressed in this article are solely those of the authors and do not necessarily represent those of their affiliated organizations, or those of the publisher, the editors and the reviewers. Any product that may be evaluated in this article, or claim that may be made by its manufacturer, is not guaranteed or endorsed by the publisher.
