# Supplementary material for: Shifting Pathways of Stimulant Use Among Individuals With Opioid Use Disorder: A Retrospective Analysis of the Last Thirty Years
Source: Front Psychiatry. 2021 Dec 20;12:786056. doi: 10.3389/fpsyt.2021.786056 (PMC8721201; doi:10.3389/fpsyt.2021.786056)
Supplement: Supplementary file 1 [file Table_1.docx]

| **Supplemental Table 1. Demographic comparison between those who used multiple substances in the year of initial drug exposure (excluded) vs. a single substance (included).** | | | | | |
| --- | --- | --- | --- | --- | --- |
|  | Excluded (n=883) | | Included (n=4,052) | | sig. (χ²) |
| **Demographics** |  |  |  |  |  |
| Female | 366 | 41.5% | 1683 | 41.9% | 0.856 |
| Racial/ethnic minority | 161 | 18.2% | 830 | 20.5% | 0.130 |
| Sexual minority | 57 | 12.2% | 258 | 13.1% | 0.614 |
| Mean age (SD) | 31.6 (7.7) | | 32.4 (7.1) | | 0.003 |
| **Urbanicity** |  |  |  |  |  |
| Urban | 407 | 47.5% | 1983 | 50.1% | 0.164 |
| Suburban | 231 | 27.0% | 1086 | 27.4% | 0.77 |
| Rural | 219 | 25.6% | 888 | 22.4% | 0.050 |
| **Regionality** |  |  |  |  |  |
| West | 164 | 18.6% | 773 | 19.1% | 0.729 |
| Midwest | 224 | 25.4% | 1075 | 26.5% | 0.477 |
| Northeast | 138 | 15.6% | 533 | 13.2% | 0.052 |
| South | 357 | 40.4% | 1671 | 41.2% | 0.658 |
| **Primary source of income** |  |  |  |  |  |
| Employed/Retired | 369 | 43.8% | 1689 | 43.5% | 0.853 |
| Public assistance | 89 | 10.6% | 479 | 12.3% | 0.155 |
| Friend/Family | 228 | 27.1% | 904 | 23.3% | 0.019 |
| Other | 156 | 18.5% | 813 | 20.9% | 0.118 |
| **Healthcare coverage** |  |  |  |  |  |
| None | 273 | 35.5% | 1358 | 39.1% | 0.061 |
| Covered under another individual | 54 | 7.0% | 170 | 4.9% | 0.017 |
| Medicare/Medicaid | 382 | 49.6% | 1686 | 48.5% | 0.583 |
| Private | 55 | 7.1% | 206 | 5.9% | 0.204 |
| VA/Military healthcare | 6 | 0.8% | 55 | 1.6% | 0.090 |
| Any healthcare coverage | 497 | 64.5% | 2117 | 60.9% | 0.061 |
| **Educational attainment** |  |  |  |  |  |
| High school or less | 521 | 59.6% | 2341 | 58.2% | 0.459 |
| Some college | 315 | 36.0% | 1505 | 37.4% | 0.436 |
| Bachelor's or higher | 38 | 4.3% | 173 | 4.3% | 0.954 |
| **Prior OUD treatment episodes** | 725 | 82.2% | 3315 | 81.9% | 0.841 |
|  |  |  |  |  |  |
|  |  |  |  |  |  |
